# Factors Determining Pakistani Medical Students’ Career Preference for General Practice Residency Training

**DOI:** 10.7759/cureus.3114

**Published:** 2018-08-06

**Authors:** Muhammad Bilal, Abdul Haseeb, Anum Mari, Mohammad Hussham Arshad, M.Raafe Ali Khan, Ayesha Ahmed, Ramsha Jeoffrey, Zainab Saleem, Muhammad Abrar Irfan, Arsalan Aamir Khan, Sana Husain, Simrah Najeeb, Amal Fuad Garib, Fatema Mustafa Attarwala, Muhammad Hasnain Mankani

**Affiliations:** 1 Medicine, Dow University of Health Sciences (DUHS), Karachi, PAK; 2 Medicine, The Wright Center for Graduate Medical Education, Scranton, USA; 3 Medicine, Ziauddin Medical University, Karachi, PAK; 4 Medicine, Aga Khan University, Karachi, PAK; 5 Medicine, Karachi Grammar School, Karachi, PAK; 6 Medicine, The Lyceum School, Karachi, PAK

**Keywords:** general practice, primary care, family practice, career choice, medical students, survey

## Abstract

Background

Few studies have explored factors affecting preference of medical students towards general practice as a career choice. We conducted a survey in Karachi across various public and private sector medical colleges to examine factors associated with students’ general practice career aspirations in Karachi, Pakistan.

Methods

From January to March 2018, we distributed a 21-item questionnaire to final year medical students in eight medical schools. The survey asked students about their top three career preferences from 19 specialty fields, their demographics and their career priorities. Multivariable logistic regression was used to determine the effect of each item.

Results

A total of 1400 responses were obtained. The top five specialty fields chosen by students with their numbers were: internal medicine, 898 (64.2%); general practice, 337 (24.1%); pediatrics, 449 (32.1%); surgery, 380 (27.2%); and emergency medicine, 243 (17.4%). The “intent to inherit existing practice” and “other academic or professional experiences prior to medical school” had a positive association with choosing general practice while “having a physician parent’’ had a negative association among the medical students demographics after adjusting for other covariates in the multivariable logistic regression. Medical students who ranked “clinical diagnostic reasoning”, “community-oriented practice”, “involvement in preventive medicine”, and “frequent patient communication” as highly important were more likely to choose general practice, whereas, “access to advanced medical fields”, “mastering advanced procedures”, and “depth rather than breadth of practice” were less likely to be associated with general practice aspiration.

Conclusion

The study’s results depicted limited interest of family medicine as a career option in graduating students, and pointed out the factors that likely influence the choice of general practice as a career are clinical diagnostic reasoning, community-oriented practice and preventive medicine.

## Introduction

Family medicine as a specialty is a multidimensional field of medicine. It deals with not only prevention and screening but also diagnosis and first-hand treatment of many acute and chronic health problems, along with a residency training in the field [1]. In many underdeveloped countries, however, general practitioners (GP) are non-specialists with no training in primary care owing to their little exposure to ambulatory and preventive care [2]. Family medicine training in Asian countries developed several decades ago, while in some nations it established more recently, for example in Japan, where recently in 2018 general practice got acknowledged as a new basic discipline [3]. In Pakistan, data regarding the details of family medicine specialists are not available [4].

The number of GPs worldwide is on a decline. Worldwide, the preference for family medicine is lacking in developed countries including the USA [5], and developing countries like Lebanon [6]. It has been observed that students did not prefer family medicine even as a backup field for their career, despite the urgent need for GPs in their country [7]. In Pakistan, there is a lack of training practice in primary care and doctors can start general practice after completing their internship, thus doctors fail to gain all the necessary skills to become competent physicians, even though training facilities have been developed adequately at Aga Khan University Karachi, King Edward University Hospital, Ziauddin University, Fatima Memorial Hospital and Liaquat University of Medical Sciences in Hyderabad [1].

Extensive research has been conducted to speculate the factors influencing medical student’s choice of a career in GP training worldwide. Factors like being female, having empathetic personality, experience in primary care during rotations are some of the important known reasons [6]. Moreover, having a rural background, no family history of medical practitioners and interest in broad scope practice careers are some of the predictors of choosing a GP career [3].

Factors that affect medical practitioners’ and medical students’ aspiration to select general practice as a specialty would be different in our context due to our own unique culture and obstacles in the field [1]. However, limited data is available for Pakistani medical students. Therefore, our study is meant to assess the factors determining preference for GP residency training among Pakistani medical students. By assessing the factors, we can help make this specialty more appealing to medical students to increase the number of skilled GPs in the future.

## Materials and methods

This cross-sectional study was conducted in several public and private medical colleges of Karachi, Pakistan. In this study, we surveyed the opinions of final year medical students towards general practice as their choice of career. We also evaluated the factors influencing the choice of family medicine specialty among medical students. Our study sample consisted of all the final year medical students of the academic year 2018. However, subjects apart from the final year medical students were excluded from the survey.

The duration of the study was three months starting from January 2018 to March 2018. The total sample size recruited in our survey was 1400. Institutions included were private medical colleges like The Aga Khan University Medical College, Baqai Medical College, Hamdard College of Medicine, Sir Syed College of Medical Science for Girls and Government medical colleges like Dow Medical College, Jinnah Sindh Medical University, Shaheed Benazir Bhutto Medical College, and Liaquat National Medical College.

We distributed a pre-tested and pre-validated 21-item questionnaire to medical students [3]. The survey asked students to select top three career options from 19 specialty fields as an outcome variable: internal medicine, pediatrics, dermatology, psychiatry, surgery, orthopedics, obstetrics and gynecology, ophthalmology, otorhinolaryngology, urology, neurosurgery, radiology, anesthesiology, pathology, clinical laboratory, emergency medicine, plastic surgery, rehabilitation, and general practice. Participant demographics (e.g., age, sex) and 14 questions regarding their career intention, using 6-point Likert scales ranging from 1 (strongly disagree) to 6 (strongly agree) were also asked.

Students were explained the objectives of the study and reassured confidentiality. All the data collected from medical students were anonymous except for the name of the medical school. Informed written consent was taken prior to data collection. The questionnaire along with the protocol of the study was approved by the Institutional Review Committee of Dow University of Health Sciences, Karachi. No conflict of interests was encountered in the entire study period. A pilot study was conducted with 30 medical students to rule out any ambiguity in the questionnaire, and the pilot study data was not included in the final analysis. Minor changes were made in the questionnaire after the pilot study had been conducted.

The Statistical Package for Social Sciences software (SPSS, version 19, IBM, Armonk, NY) was used for data analysis and data entry. Descriptive biostatistics was applied. Chi-square test was used to analyze the difference in the proportions. The p-value of less than 0.05 was considered statistically significant. Univariable and multivariable logistic regressions were used for each demographic factor and career intentions in terms of odds ratio and at 95% confidence intervals. The outcome variables were divided into two categories on the basis of whether the general practice was chosen in the first three career choices or not. Variables such as gender, any other work or field experience prior to medical school, having physician-parent and plan to inherit other’s practice were taken as binary while birthplace as nominal and age as a continuous variable. The responses to the Likert scale of 14 career intention questions were treated as continuous.

## Results

A total of 1400 responses were obtained at a 100% response rate. All of them were included in the final statistical analysis. Table 1 highlights demographic characteristics of GP and non-GP candidates among Pakistani medical students. This includes distribution of the participants’ sex, age, birthplace, job or other field experiences prior to medical school, physician-parent, plan to inherit other’s practice and career priorities. The survey participants had a median age of 24, 33% were male, 32.2% had a physician-parent, and 11.5% had a positive intent to inherit an existing practice. The top five specialty fields are chosen by students and their numbers were: internal medicine, 898 (64.2%); general practice, 337 (24.1%); Pediatrics, 449 (32.1%); surgery, 380 (27.2%); and emergency medicine, 243 (17.4%).

**Table 1:** Characteristics of GP candidates and non-GP candidates among Pakistani medical students. ^a^Please select one of the following options which best describes your thoughts regarding your career priorities. (1 = strongly disagree, 6 = strongly agree). GP: General practitioner

	Total N = 1400	GP N = 449	Non-GP N = 951
Demographics; no. (%) of students			
Age, median (range), years	24 (23-27)	24 (23-27)	24 (23-25)
Sex (male)	462 (33)	54 (12)	408 (42.9)
Hometown			
Urban	298 (21.3)	79 (17.6)	223 (23.4)
Relatively urban	315 (22.5)	112 (25.1)	217 (22.8)
Relatively rural	442 (31.6)	141 (31.6)	292 (30.8)
Rural	345 (24.6)	117 (25.7)	219 (23)
Other academic or professional experiences prior to medical school	317 (22.8)	125 (28)	192 (20.2)
Physician parent	450 (32.2)	118 (26.4)	332 (34.9)
Intent to inherit existing practice	161 (11.5)	64 (14.1)	97 (10.2)
Career priorities^a^; mean (SD)			
Mastering advanced procedures	4.81 (1.00)	4.60 (1.03)	4.93 (0.97)
Work life balance	4.87 (0.93)	4.91 (0.89)	4.87 (0.95)
Frequent patient communication	4.80 (0.89)	5.02 (0.82)	4.72 (0.90)
Opening own clinic	3.32 (1.34)	3.48 (1.34)	3.26 (1.35)
Involvement in preventive medicine	4.08 (1.13)	4.40 (1.04)	3.90 (1.14)
Involvement in terminal care	3.76 (1.15)	4.06 (1.03)	3.63 (1.17)
Acute care rather than chronic care	4.10 (1.06)	3.96 (1.02)	4.20 (1.07)
Not treat patients with psychosocial problems	2.75 (1.19)	2.50 (1.14)	2.84 (1.19)
Income	4.16 (1.00)	4.04 (1.08)	4.27 (0.96)
Access to advanced medical fields	4.29 (0.98)	4.06 (0.97)	4.41 (0.96)
Clinical diagnostic reasoning	4.30 (1.00)	4.60 (0.95)	4.14 (0.99)
Depth rather than breadth of practice	3.96 (1.02)	3.63 (0.97)	4.19 (1.03)
Involvement in global health	3.37 (1.13)	3.43 (1.13)	3.31 (1.12)
Community-oriented practice	4.09 (1.05)	4.45 (0.98)	3.80 (1.03)

Moreover, Table 2 indicates the crude and adjusted odds ratios of characteristics of GP candidates among final year medical students of Pakistan. In univariate analysis, we found those born in urban areas and suburban areas, compared to rural areas, and having a physician-parent, both had significant effects on students’ general practice career preference (Table 2). The “intent to inherit existing practice” and “other academic or professional experiences prior to medical school” had a positive association with choosing general practice while “having a physician-parent” had a negative association among the medical students' demographics (Table 2). As for the 14 career priorities, seven positive influences and six negative influencers were identified in crude (or univariate) analyses (Table 2). After adjusting for other covariates in the multivariable logistic regression, seven positive influencers and six negative influencers remained significant. Medical students who ranked “clinical diagnostic reasoning”, “community-oriented practice”, “involvement in preventive medicine”, and “frequent patient communication” as highly important were more likely to choose general practice, whereas, “access to advanced medical fields”, “mastering advanced procedures”, and “depth rather than breadth of practice” were less likely to be associated with general practice aspiration (Table 2).

**Table 2:** Crude and adjusted odds ratio of characteristics of GP candidates among Pakistani medical students. Dependent variable: Whether general practice was included in up to three choices (1) or not (0) *Statistically significant at an alpha level of 0.05. GP: General practitioner

OR (95% CI)	Crude	Adjusted
Demographics		
Sex (male)	0.92 (0.70-1.25)	0.84 (0.64-1.15)
Hometown		
Urban	0.85* (0.60-0.98)	0.79* (0.54-0.90)
Relatively urban	0.88* (0.75-0.97)	0.82* (0.70-0.92)
Relatively rural	1.25 (0.90-1.54)	1.20 (0.85-1.49)
Rural	1.21 (0.90-1.60)	1.15 (0.80-1.50)
Other academic or professional experiences prior to medical school	1.22* (1.12-1.34)	1.20* (1.10-1.30)
Physician parent	0.70* (0.50-0.90)	0.64* (0.45-0.85)
Intent to inherit existing practice	1.40* (1.10-1.70)	1.60* (1.20-2.00)
Career priorities		
Mastering advanced procedures	0.70* (0.60-0.80)	0.80* (0.72-0.88)
Work life balance	1.04 (0.90-1.15)	0.95 (0.80-1.10)
Frequent patient communication	1.60* (1.20-2.00)	1.80* (1.50-2.10)
Opening own clinic	1.20* (1.05-1.35)	1.28* (1.14-1.42)
Involvement in preventive medicine	1.60* (1.25-1.85)	1.35* (1.20-1.50)
Involvement in terminal care	1.40* (1.28-1.68)	1.15* (1.05-1.30)
Acute care rather than chronic care	0.78* (0.60-0.96)	0.72* (0.60-0.84)
Not treat patients with psychosocial problems	0.76* (0.60-0.90)	0.80* (0.65-0.95)
Income	0.82* (0.73-0.93)	0.88* (0.80-0.96)
Access to advanced medical fields	0.68* (0.50-0.86)	0.85* (0.71-0.98)
Clinical diagnostic reasoning	1.60* (1.40-1.80)	1.65* (1.30-1.95)
Depth rather than breadth of practice	0.60* (0.40-0.80)	0.68* (0.48-0.88)
Involvement in global health	1.20* (1.10-1.30)	1.22* (1.08-1.36)
Community-oriented practice	1.77* (1.55-2.05)	1.35* (1.05-1.65)

## Discussion

The choice of specialty by medical students is a complex decision-making process involving several factors. This process is as important for the students as it is for the health care system of the community since it has significant implications for both [8]. Therefore, the career choices of medical students are of increasing concern to government and health institutions who collectively make an effort to provide the right balance of medical professionals in each field to meet the needs of their communities [9]. The trend away from family medicine has lasted for several years, and the literature is full of strategies aimed at encouraging students to opt for primary care careers [10-14]. In fact, developing strategies to encourage medical graduates to begin a career in primary care is now being recognized as a global challenge [15]. The gap is attributable to the lack on the part of the health system and medical education institutions that do not reinforce the need for general practitioners in the country and the increasing exposure to other sub-specialties means that they become a priority over family medicine in the medical students’ mind [16].

Our study was a cross-sectional survey of final year medical students’ in different public and private institutions of Karachi, Pakistan. The study’s results depicted limited interest of family medicine as a career option in graduating students and pointed out the factors that likely influence this by recording the student responses in 14 career priority questions that included some questions regarding their family careers and their prior professional exposure. As a matter of fact, our study results showed general practice, while still under the top five specialty list, was not considered a principal career choice, as was also found in another study [17]. The medical students who ranked “clinical diagnostic reasoning”, “community-oriented practice”, “involvement in preventive medicine”, and “frequent patient communication” as highly important were more likely to choose general practice, whereas, those who selected “access to advanced medical fields”, “mastering advanced procedures”, and “depth rather than breadth of practice” were less likely to be associated with general practice aspiration. However, the present study should not be considered and viewed as a disappointment of the health institutions’ programmes, rather, it is a baseline study indicating that increased efforts are required by health care professionals in the field to bring about a change at an academic and national policy level.

The “intent to inherit existing practice” and “other academic or professional experiences prior to medical school” had a positive association with choosing general practice while “having a physician-parent” had a negative association among the medical students demographics, which is consistent with existing literature from North America and Europe [18, 19]. The reason for this may be the esteemed perception of specialties other than general practice in physician parents’ mind that could negatively influence their children [3]. Literature has proven that having friends and family in general practice is positively associated with the choice of GP [20], however, our study did not investigate that. The positive association between the “intention to inherit existing practice’’, “clinical diagnostic reasoning”, “community-oriented practice”, “involvement in preventive medicine and global health”, “frequent patient communication” and general practice choice was consistent with the findings of Japanese survey [3], whereas, “access to advanced medical fields”, “mastering advanced procedures”, and “depth rather than breadth of practice”, and “income’’, were less likely to be associated with general practice aspiration as described in another study [21]. Of the 14 career priorities, “Community-oriented practice” was the strongest predictor of general practice choice. A student’s community orientation has been shown to predict primary care career preference and was a factor affecting family medicine career choice in Japan [22].

The study also found those born in urban and suburban areas, rather than rural, were more likely to opt for general practice, which is inconsistent with some other studies [23-25]. Being a female was not associated with the choice of general practice as per the results of our study, however other studies have found a significant association between the two [3]. While family medicine preference at an entry to medical school is known to increase the probability of choosing a family medicine career [19, 26], this item was not included in our study.

The 100% response rate was the strength of our study and indicates that this topic was important to the newly graduating final year medical students. Despite the thorough development of the questionnaire and relatively large sample size, our study has several limitations. Our outcome measurement was career aspiration only during the final year of medical school, the actual enrolment in general practice residency and subsequent retention rate should also be considered in the future as after graduation there is maybe a shift from one preference to another. Because our study employed convenience sampling of medical schools, the representativeness of our study results to the general medical student population would be limited and thus, the study results may not be generalizable to other provinces of Pakistan or other countries. To our knowledge, however, this is the first and largest survey conducted across medical schools of private as well as the public sector of Karachi.

## Conclusions

The study’s results depicted limited interest of family medicine as a career option in graduating students. Factors that likely influence the choice of general practice as a career were found to be clinical diagnostic reasoning, community-oriented practice, and preventive medicine. While access to advanced medical fields, mastering advanced procedures’ and depth rather than breadth of practice were less likely to be associated with general practice aspiration. Further survey is recommended to include a greater number of medical faculties throughout the country to better understand the factors that likely influence students’ decision for choice of specialty in residency training which would in turn help determine the correct policies to facilitate general practice career choice by future doctors.
